# Supplementary material for: Voluntary Reduction of Social Interaction during the COVID-19 Pandemic in Taiwan: Related Factors and Association with Perceived Social Support
Source: Int J Environ Res Public Health. 2020 Oct 31;17(21):8039. doi: 10.3390/ijerph17218039 (PMC7672562; doi:10.3390/ijerph17218039)
Supplement: Supplementary file 1 [file ijerph-17-08039-s001.zip › ijerph-969307-supplementary.docx]

Supplementary Table S1 Measures used in this study

| Measures | Items | Response scale | Scoring | |
| --- | --- | --- | --- | --- |
| Worry about contracting COVID-19 | Item 1: If you were to develop flu-like symptoms tomorrow, would you be | 1 = not at all worried, 2 = worried less than normal, 3 = about the same, 4 = worried more than normal, 5 = extremely worried | Divided by 5 | Summed to obtain a score. The median total score was used as the cutoff. |
|  | Item 2: In the past one week, have you ever worried about catching COVID-19? | 1 = no, never think about it, 2 = think about it but it didn't worry me, 3 = worried me a bit, 4 = worried me a lot, 5 = worried about it all the time | Divided by 5 |  |
|  | Item 3: Please rate the current level of your worry towards human swine flu: | Score ranged from 1-10 (1 = very mild, 10 = very severe) | Divided by 10 |  |
|  | Item 4: How likely do you think it is that you will contract COVID-19 over the next 1 month? | 1 = never, 2 = very unlikely, 3 = unlikely, 4 = evens, 5 = likely, 6 = very likely, 7 = certain | Divided by 7 |  |
|  | Item 5: What do you think are your chances of getting COVID-19 over the next 1 month compared to others outside your family? | 1= not at all, 2 = much less, 3 = less, 4 = evens, 5 = more, 6 = much more, 7 = certain | Divided by 7 |  |
| General anxiety | Agreement on 10 statements: You feel rested /content/comfortable/relaxed/pleasant/anxious/nervous/jittery/“high strung”/over-excited and “rattled” | 1 = not at all, 2 = sometimes, 3 = moderately so, 4 = very | Summed to obtain a score. The median total score was used as the cutoff. | |
